# The Bromides

**Published:** 1883-07

**Authors:** 


### THE BROMIDES.

Are there important points in which these useful salts differ among themselves as to their therapeutical powers? Careful experiments show that they do—that when a bromide is indicated there are conditions often present which require a nice, but easily attainable discrimination as to which one is best for the case.

The empirical fashion is to exhibit potassium bromide in almost all occasions, as if this salt is the exact physiological synogist of each of the others.

It is high time for the profession to exercise more precision in this matter. The following *bromidian laws* are worth some study:

1st. The continuous use of any of the bromides will produce gastric catarrh.

2d. The bromides are exceedingly diffusible, passing quickly into the blood and system.

3d. The physiological effect of all the bromides is to depress, but in very different degrees, heart action, temperature and respiration.

4th. All the bromides are pronounced anaphrodisiacs.

5th. The long continued use of the bromides will produce pallor, anæmia, dilated pupils, acne on face and forehead, fetid breath, feeble heart action, breathlessness and accelerated pulse on the least exertion, feebleness of mind and death of all sexual desire.

6th. As depressors of pulse, heat and respiration, the bromides of ammonium and potassium stand highest; the bromides of lithium and sodium lowest.

7th. Of all the bromides, the potassium has the highest toxic power; the sodium least.

8th. In controlling reflex action, ammonium bromide is first, potassium second, lithium third and sodium fourth.

9th. If the letheal effect of the bromides is desirable, while on the other hand, their depressing powers are contra-indicated, Brown-Sequard advises the simultaneous exhibition of digitalis or even of theine.

10th. The same author advises the combined administration of sodium, ammonium and potassium bromide in epilepsy.

11th. Clinically, the dangers of cardiac depression from potassium bromide and chloral are fully recognized.

12th. Of the bromides, chlorides and iodides of sodium and potassium, those of potassium are ten times more potent than those of sodium.

14th. With these advantages, the general verdict of clinical experience is, that as a hypnotic, sodium bromide is the sedative above all others to be chosen.

